# Dimerization Effects and Negative Strain Energy in Silicon Monosulfide Nanotubes

**DOI:** 10.3390/nano13233033

**Published:** 2023-11-27

**Authors:** Tomás Alonso-Lanza, Faustino Aguilera-Granja, Andrés Ayuela

**Affiliations:** 1 Centro de Física de Materiales CFM-MPC CSIC-UPV/EHU, 20018 San Sebastián, Spain; tomas_alonso001@ehu.eus; 2 Instituto de Física, Universidad Autónoma de San Luis de Potosí, San Luis Potosí 78000, Mexico; faustino@ifisica.uaslp.mx; 3 Donostia International Physics Center (DIPC), 20018 San Sebastián, Spain

**Keywords:** inorganic nanotubes, silicon monsulfides, isoelectronic phosphorene, dimerization, negative strain energy

## Abstract

We report on the construction and characterization of silicon monosulfide nanotubes that were obtained by rolling up two-dimensional materials isoelectronic to phosphorene in the recently discovered layered *Pmma* and β phases. We relaxed and studied the nanotube structures using computational methods within density functional theory (DFT). We found that the nanotubes with a thick *Pmma* layer remain stable at room temperature, and their electronic properties depend on their diameters. Small-diameter nanotubes display metallic character, while nanotubes with increasing diameter show semiconducting ground states due to the dimerization in the silicon–silicon distances that opens a gap, leading to interesting optical properties in the near-infrared region. Furthermore, we discovered β SiS monolayer nanotubes having negative strain energies, similar to the well-known imogolite inorganic nanotubes. The combined thermal stability, compelling optical properties, and diverse applications of these silicon monosulfide nanotubes underscore the demand for novel synthesis methods to fully explore their potential in various fields.

## 1. Introduction

**Figure 1:**
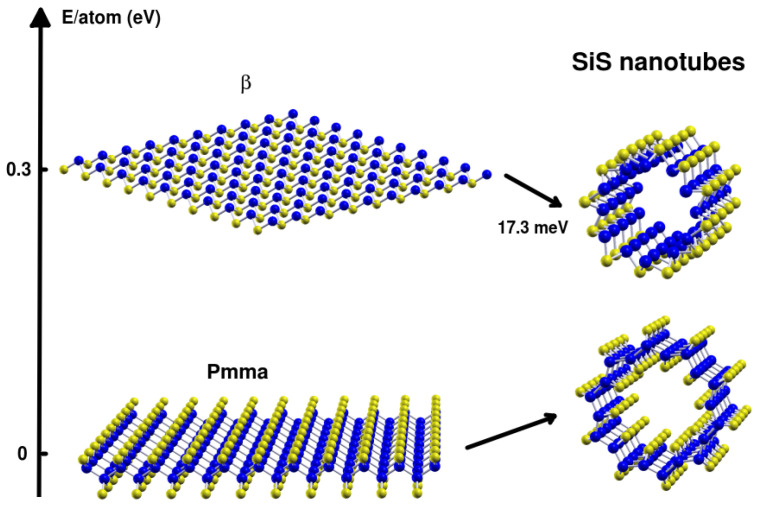
Exploring the energy landscape of SiS nanotubes by rolling up stable two-dimensional layers. Si atoms are colored in blue, and S atoms in yellow. Different nanostructures are compared according to their energy per atom.

Inorganic nanotubes have atracted significant attention since the discovery of WS${}_{2}$ nanotubes in 1992 [1], followed by other examples such as MoS${}_{2}$ [2] and imogolite nanotubes [3]. Furthermore, inorganic boron nitride nanotubes have been synthesized experimentally [4,5], exploiting their isoelectronic nature to carbon nanotubes. Then, researchers have explored a wide range of materials for nanotube formation, including various transition metal dichalcogenides (TMDCs), as well as compounds consisting of three or four elements, such as misfit layered compounds [1,6,7,8]. These misfit compounds consist of alternating slabs of hexagonal layered compounds, such as disulfides, and monosulfides in other structural phases. As the development of two-dimensional materials continues, the search for new materials that can be rolled into nanotubes is ongoing [9,10,11,12,13,14]. Recently, phosphorene layers were discovered, and the corresponding nanotubes have already been studied [9,10,15]. The next potential candidate layers to be investigated seem to be the two-dimensional materials isoelectronic to phosphorene, such as those based on silicon-sulfide SiS [16,17,18]. Several two-dimensional silicon monosulfide layers have been reported [16,17,18], which can be simplified into three monolayer structures and two thicker, more stable structures. Of these, two monolayer hexagonal structures were found analogous to the black and blue phosphorus bulk [16,19], while the third has a rombohedral unit cell that was shown to be stable [18]. Thicker SiS nanolayers have also been investigated [17], revealing very stable phases such as *Pmma* structures, followed by silicene ones being both cubic structures. We aimed here to investigate the properties of one-dimensional SiS nanotubes by rolling the two key bi-dimensional SiS nanostructures, as shown in Figure 1.

In this paper, we present a comprehensive study on the stability, electronic, and optical properties of silicon monosulfide (SiS) nanotubes. We focused on the *Pmma* phase, which is the most stable of SiS layers, to start building our nanotubes. To ensure the stability of SiS nanotubes, we performed molecular dynamics simulations at room temperature. Interestingly, we found that the nanotubes built from the β phase are also stable at higher energy and have negative strain energy, a feature previously reported for imogolite nanotubes. We also investigated the electronic and optical properties for SiS nanotubes and found potential applications in optoelectronic devices, particularly in the infrared region [17].

## 2. Computational Details

We employed the Spanish Initiative for Electronic Simulations with Thousands of Atoms (SIESTA) method [20] to perform density functional theory (DFT) calculations of silicon monosulfide nanotubes. We used the Perdew–Burke–Ernzenhof form of the generalized gradient approximation (GGA) for the exchange and correlation functional [21]. A meshcutoff of 250 Ry and an electronic temperature of 25 meV were employed. We sampled the one-dimensional Brillouin nanotube zone using 16 k points. To avoid interaction with the images, the cells were given vectors around 40 Å in the two directions perpendicular to the tube axis. The atomic positions and the unit cell parameters were converged until the atomic forces were below 0.006 eV/Å. The atomic cores were described by non-local norm-conserving Troullier–Martins [22] pseudopotentials, which were factorized in the Kleinman–Bylander form. We considered the same silicon and sulfur pseudopotentials and the basis composed of double zeta plus polarization orbitals as those used for SiS monolayers in Ref. [18]. To ensure the reliability of our results, we repeated test calculations with the VASP code within the PBE formalism for exchange and correlation with the projected augmented wave method (PAW) [18,23,24,25]. We also performed molecular dynamics calculations using the SIESTA method with a Nose thermostat, a Nose mass of 10 Ry fs${}^{2}$, and a time step of 1 fs to ensure the stability of the relaxed structures with temperature [26,27,28].

## 3. Results and Discussion

We investigated two different types of SiS nanotubes, illustrated in the diagram of Figure 1. Firstly, we examined *Pmma* nanotubes obtained by rolling up the most stable SiS layers, the thick *Pmma* planar phase. Secondly, we analyzed nanotubes built from the β monolayer. It is noteworthy that the β nanotubes exhibit negative strain energy, a fact shared with imogolite nanotubes, indicating a preference for curved surfaces. Therefore, we presented our findings into two main parts: one for *Pmma* nanotubes and the other for β nanotubes.

### 3.1. Pmma SiS Nanotubes: Dimerization Effects

The most stable two-dimensional structure of silicon monosulfide (SiS) is the *Pma2* phase, as reported recently, followed closely by the *Pmma* phase [17]. The *Pma2* structure is slightly more stable than the *Pmma* one due to small structural distortions of sulfur atoms, being stabilized in a larger unit cell. However, this effect of structural distortions was negligible when considering nanotube calculations, as the deformation imposed by the tube curvature takes precedence. Therefore, no expected distortions between the sulfur atoms were found in the nanotubes built with the large *Pma2* unit cell. We tested *Pma2* nanotubes, and they relaxed to be *Pmma* ones. That is why we next discuss results on the *Pmma* SiS nanotubes.

**Figure 2:**
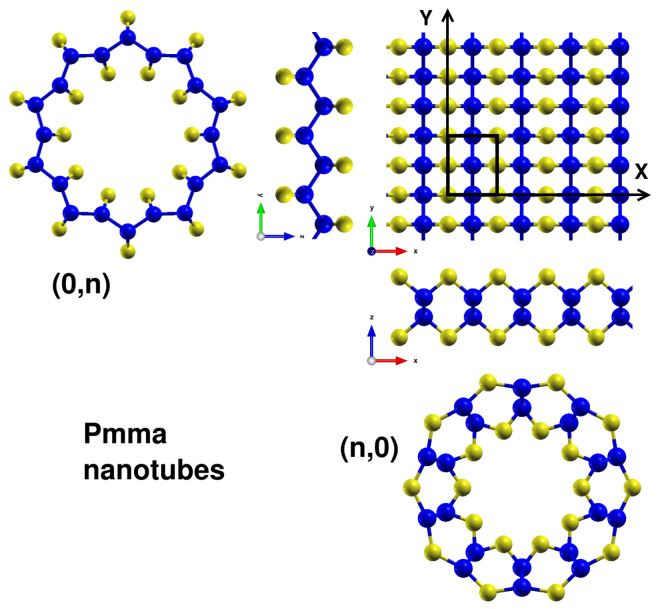
Rolling directions of *Pmma* SiS layer for the construction of (n,0) and (0,n) nanotubes, with the highlighted *Pmma* unit cell using a black rectangle.

The rectangular unit cell used to roll the *Pmma* layer and build the nanotubes is depicted in Figure 2, with examples of the initial geometries for the (n,0) and (0,n) *Pmma* nanotubes. Some of the (n,0) tubes with small curvature experienced large deformations during structural relaxations, having significant reconstructions into silicene-like layers, as discussed in Appendix A.1. Moreover, the relaxed structures of (n,0) tubes are less stable than those of the (0,n) tubes. For instance, the energies per atom for nanotubes (n,0) with a radius around 7.1 Å are at least 50 meV/atom higher than for the (0,n) counterparts. Therefore, we focused our discussion on the SiS nanotubes (0,n), as they maintain their initial structure after rolling the *Pmma* layer, and the structural relaxations converge smoothly to the final ground-state geometries.

**Figure 3:**
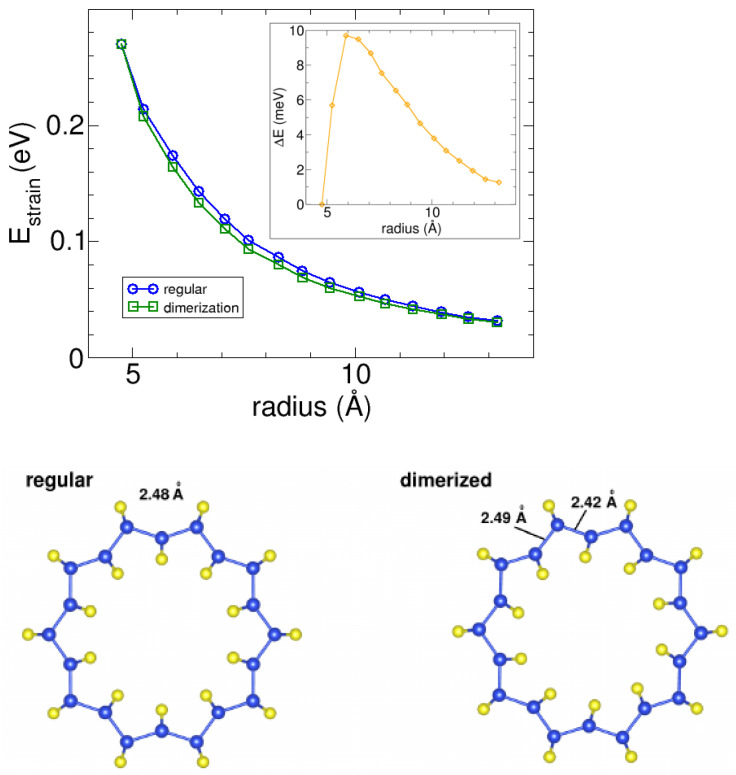
Relationship between the strain energy and the average radius for (0,n) *Pmma* SiS nanotubes with regular and dimerized structures. The inset shows the difference in energy per atom resulting from the dimerization with respect to the more regular structures. Structures below show the the regular and dimerized structures using the (0,10) nanotube as an example.

We first studied the strain energy of the nanotubes (0,n), which reflects the energy differences between the nanotubes and the *Pmma* phase due to the nanotube curvature. Specifically, we calculated the strain energy as $E_{strain}=E_{nanotube}-E_{Pmma}$, where we determined the energy *E* per atom for both the nanotubes and the flat Pmma phase using the same computational details. Figure 3 shows the variation of the strain energy as a function of the average radius of the (0,n) nanotubes. Note that the nanotube average radius was determined as the average value of the radii of the different types of silicon and sulfur atoms. Our results show that the strain energy for (0,n) nanotubes is positive so that the flat *Pmma* phase is more stable than the tubes. As the diameter of the tube increases, the strain energy appears to converge towards zero. Anyhow, the values obtained for $E_{strain}$ are consistent with those found for carbon [29,30] and inorganic nanotubes [31] that have been synthesized experimentally to date.

Furthermore, the (0,n) nanotubes after relaxations can exhibit two distinct structures for each size, with energy differences of a few meVs per atom. We observed two curves for the SiS nanotubes (0,n) corresponding to regular and dimerized structures. The inset of Figure 3 illustrates that the dimerized nanotubes are more stable than the regular ones by 1–10 meV per atom. In the regular structures, the distance between silicon atoms across the cross-section is constant, while in the dimerized nanotubes, the silicon–silicon distances alternate between short and long distances across the tube. This dimerization effect is small, with differences of 0.03–0.07 Å between the silicon–silicon distances across the tubes. The effect starts at a radius of 5 Å, reaches a maximum, and then decreases for larger radii. In addition, we carried out molecular dynamics simulations that confirm the stability of the (0,n) nanotubes in the Pmma phase at room temperature. Further details on these simulations are provided in Appendix A.2.

The source of distortions ultimately lies in symmetry breaking. Geometric distortion is determined by changes in transverse distances, which lead to a breakdown of symmetry. This, in turn, results in the mixing of states that previously intersected at the Fermi level, eventually leading to gap opening and increased energy per atom, subsequently making the distorted nanotubes more stable. We analyzed these quantities, such as dimerization distances, gaps, and bands, individually to enhance our understanding on the origin of distortions.

**Figure 4:**
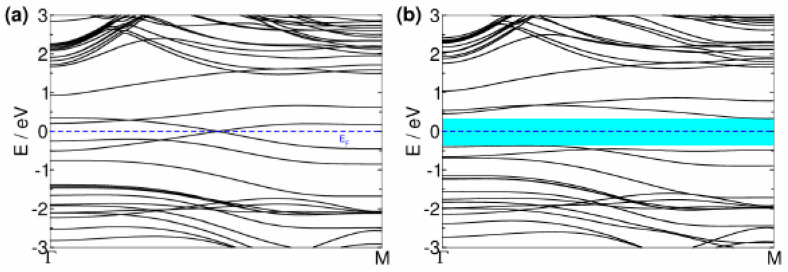
Band structure of *Pmma* SiS tubes for (**a**) regular and (**b**) dimerized structures of the (0,10) nanotube.

**Figure 5:**
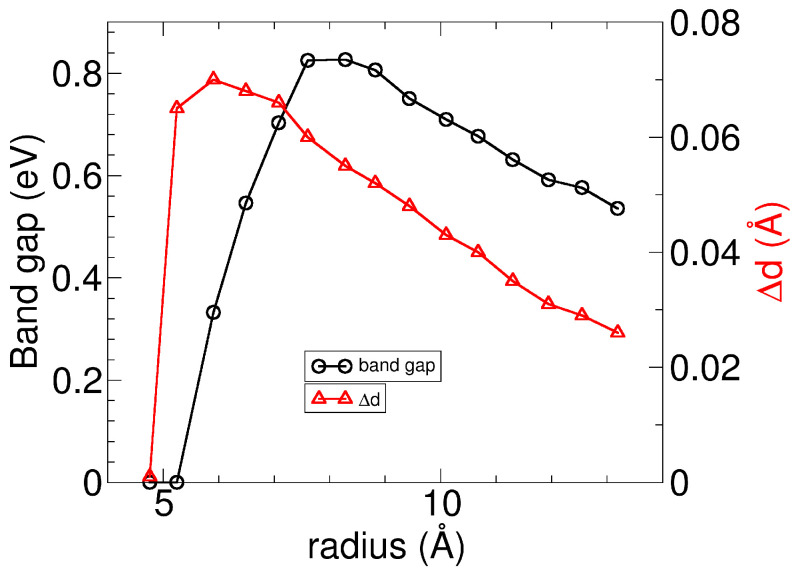
Variation of band gaps and Si–Si distances with respect to radii for the dimerized (0,n) nanotubes of *Pmma* SiS. Gaps of the dimerized tubes are below the one of the *Pmma* layer (calculated about 1.22 eV using hybrid calculations [17]).

The Si–Si dimerization has a significant impact on the electronic structure of SiS nanotubes. As shown in Figure 4a, the band structure of a regular (0,n) nanotube reveals that around the Fermi energy, the hole bands are mainly localized in the Si–S–Si and Si–Si bonds, becoming unoccupied, while the electron bands of Si dangling bonds are partially occupied. The two bands cross the Fermi energy at an intermediate point, making the regular (0,n) nanotubes metallic. However, the Si–Si dimerization opens a non-negligible gap in the electronic structure of the regular (0,n) nanotubes, as shown for the dimerized SiS nanotube in Figure 4b, and the dimerized nanotubes turn into semiconductors. Using PBE is acceptable, but it is reported to underestimate the gap [32,33]. Note that the gap opening due to dimerization happens even when we use hybrid functionals, as discussed in Appendix B. The variation of the band gap of the dimerized SiS nanotubes as a function of the tube size is displayed in Figure 5. The band gap increases with the radius, reaching a maximum value before decreasing monotonically. The maximum in the band gaps is shifted to a larger radius than the maximum in the geometry dimerization, indicating that the gap opens after the dimerization in distances. Appendix A.3 includes additional details on the analysis of the projected density of states in atoms, given for the dimerized (0,10) nanotube. As expected, sulfur contributes with *s* states at deep energies and with *p* states at higher energies. Silicon atoms also have *s* states at deep energies and *p* states near the Fermi energy and show a non-negligible overlap with *d* states due to the nanotube curvature. It should be noted that the *p–d* Si hybridization is negligible in the *Pmma* layer.

**Figure 6:**
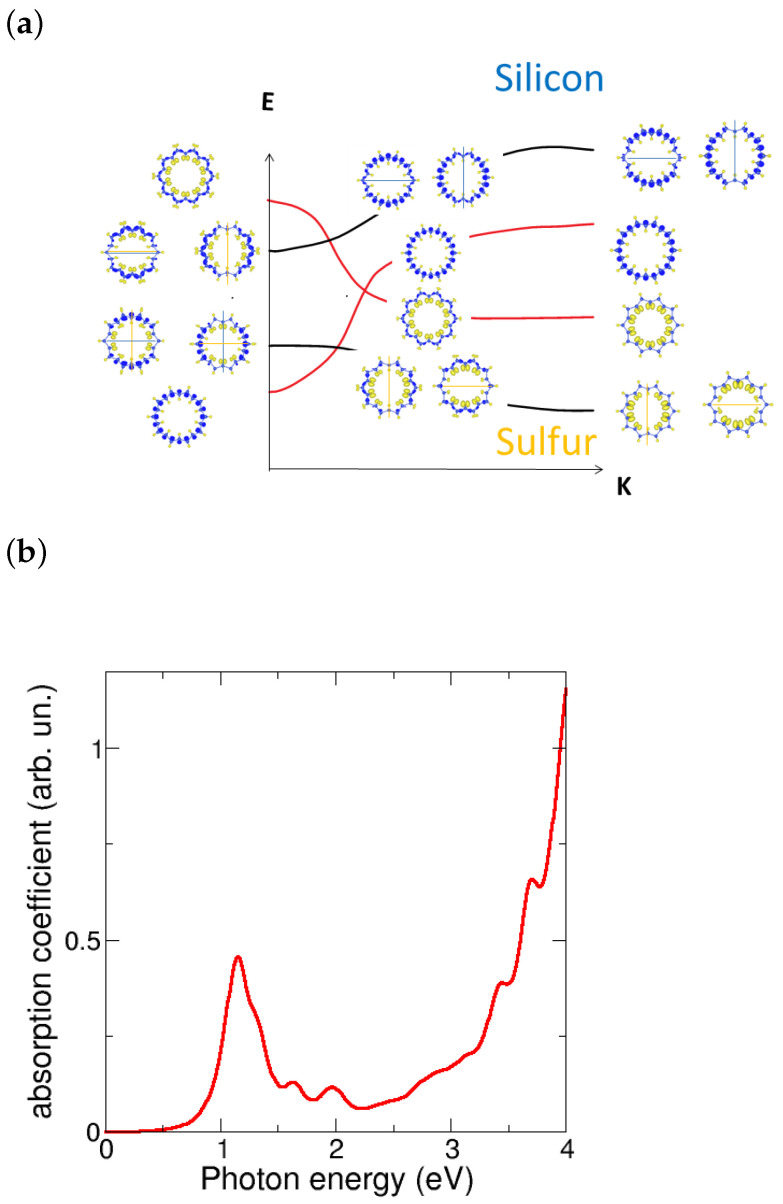
(**a**) Scheme of partial charge densities decomposed in bands and sites at selected *k* points (0, 0.5, 1 from left to right) along the regular (0,10) tube. The bands around the Fermi energy exhibit different symmetries, characterized by the presence or absence of nodal planes. The bands near the Fermi energy without nodes exhibit different characters of sulfur and silicon, which become mixed due to the Si–Si dimerization and the gap opening. (**b**) Calculated absorption coefficient for the (0,10) dimerized nanotube. It is worth noting the significant absorption observed in the UV–visible range, along with a prominent peak responsible for emission in the infrared range.

To provided a more detailed explanation of the bands crossing around the Fermi energy and the gap opening, we have studied the partial charge densities and developed a scheme as follows. Figure 6a displays the band scheme around the Fermi energy for the (0,10) metallic tube, which includes the partial charge densities decomposed in bands and sites at selected *k* points such as k= 0, 0.5, and 1 in reciprocal lattice units. The bands that cross at $E_{f}$ are once degenerated and shown in red color. The lower band at k=0 in the figure has mainly Si character, while the upper one has S character; both bands are nodeless, indicating a global rotation symmetry in the nanotube with $m_{l}=0$. The bands next to $E_{f}$ at k=0 have Si and S character for lower and higher energy, respectively; they are doubly degenerated and show nodal planes, which correspond to symmetries in tubes with the angular momentum $m_{l}=+1,-1$. Since the bands near the Fermi energy have different symmetries $m_{l}=0,\pm 1$, extra optical activity between them is expected. The band crossing enables optical transitions between the bands near the k=0 point and the *M* point. By looking at the above gap values, we found that the emission of these nanotubes would fall into the near-infrared regime.

The optical properties of the Pmma SiS layers were found promising for applications in the visible regime [17]. We then calculated the complex dielectric function within the independent particle approximation and obtained the adsorption coefficient α(ω) from the real and imaginary parts of the dielectric function. Figure 6b shows the optical absorption coefficient for the (0,10) dimerized nanotube. Although the Pmma tubes show indirect gaps, the flatness of the bands results in non-negligible absorption, which is caused by the gap opening due to dimerization. This enables optical transitions between the top valence and low conduction bands. While SiS layers are more suitable for photovoltaic applications in the visible range, SiS nanotubes are more interesting for emission purposes in the infrared regime even in the far atmospheric window. Furthermore, the SiS nanotubes show intriguing emission properties in the infrared regime, particularly within the atmospheric window. This makes them appealing for applications requiring radiative cooling or infrared emission [34]. Thus, the versatility of SiS layers and tubes nanomaterials enables their utilization in a wide range of applications, harnessing absorption in the visible range and emission in the infrared atmospheric window.

### 3.2. Nanotubes Rolling up β SiS Monolayers: Negative Strain Energy

**Figure 7:**
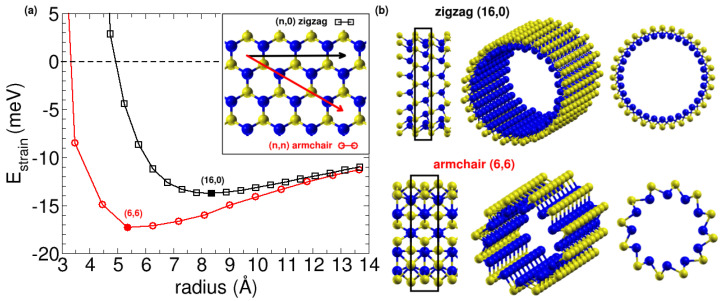
(**a**) Strain energy as a function of the average radius for armchair and zigzag types of β SiS nanotubes. The inset shows the rolling directions in the β SiS monolayer that result in the two types of β SiS nanotubes. (**b**) Different perspectives of the atomic structures of the most stable β SiS nanotubes, the zigzag (16,0) and armchair (6,6) nanotubes.

Our initial calculations for β SiS nanoribbons indicated that the corresponding nanotubes could potentially be stable. Upon relaxation of the atoms, the SiS β nanoribbons were found to curve spontaneously, with the larger S atoms located on the outside and the smaller Si atoms on the inside. Therefore, we further investigated the stability of the SiS nanotubes by rolling the β SiS layer into two types of chiralities—armchair (n,0) and the zigzag (n,n), as shown in the inset of Figure 7a. We compared the energy per atom of the relaxed nanotubes with that of the flat β phase. The strain energy per atom ( $E_{strain}=E_{nanotube}-E_{\beta }$) for both armchair and zigzag nanotubes is shown as a function of the average nanotube radius in Figure 7a. The strain energy is found to be negative below that of the β monolayer for increasing average nanotube radius. This finding indicates that the β SiS nanotubes are more stable than the β monolayer due to their spontaneous curvature, a phenomenon that has been also observed and studied in imogolite nanotubes [3,12,13].

We next examined the dependence of strain energy on the size of the nanotube, focusing on the relative stability within armchair and zigzag nanotubes. We noted that for the β SiS tubes, the (6,6) armchair nanotube is the most stable, with a radius of 5.36 Å and a strain energy of 17.3 meV per atom lower than the flat β layer. For zigzag nanotubes, the minimum occurs at a larger radius of 8.36 Å and is 13.7 meV per atom still below the flat β phase. The geometries of the two minima are illustrated in Figure 7b. Interestingly, in both types of nanotubes, the small silicon atoms are located inside and the sulfur atoms are outside. It is natural for S to be on the outside due to its higher electronegativity, which results in it being negatively charged and segregated from Si atoms. Additionally, we investigated the stability of inverse nanotubes by swapping the positions of S and Si atoms and found that they are indeed less stable than the β monolayer. We fitted the evolution of the strain energy with nanotube size using an equation that relates the strain energy to the nanotube radius: $E_{strain}=\frac{a}{R^{2}}∼\frac{Yh^{3}}{R^{2}}$, where *a* is a parameter proportional to the product of the Young modulus *Y* and the nanotube thickness *h* to the third. This equation works for most of the nanotubes, such as the (0,n) Pmma nanotubes studied above. However, it cannot be directly applied to nanotubes with negative strain energy, such as imogolite nanotubes [3,12] and β SiS nanotubes studied in this section. An extra term accounting for the difference in surface energy between inner and outer surfaces is needed because the tube is not symmetric. The equation was thus replaced by $E_{strain}=\frac{a}{R^{2}}+\frac{b}{R}∼\frac{Yh^{3}}{R^{2}}+\frac{\Delta \sigma h}{R}$, where *b* is another parameter proportional to the product of the surface tensions Δσ and the thickness *h*. We had checked that this equation fits the strain energy for the β SiS nanotubes, with fitting parameters a=0.64 and b=−0.2087. Taking *h*∼ 1.4 Å we obtained Y=37.28 GPa. To obtain negative strain-energy nanotubes, it is necessary that the flat surface is not symmetric on both sides, which is the case for the β SiS monolayer.

**Figure 8:**
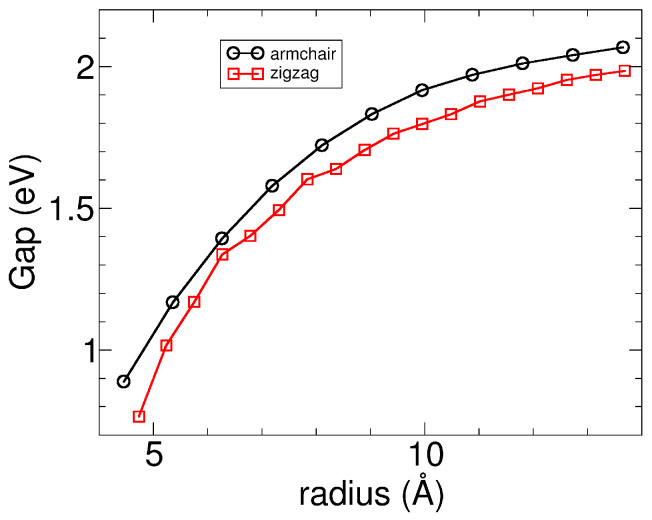
Band gaps as a function of the radii of the armchair and zigzag β SiS nanotubes.

*Electronic properties.* The trends of band gaps with the nanotube radius are presented in Figure 8. It was observed that the β SiS nanotubes exhibit semiconductor behavior and the gaps increase gradually for both armchair and zigzag nanotubes. For armchair nanotubes, the band gap lies in the range of 0.90–2.07 eV spanning from (3,3) to (15,15) sizes. On the other hand, for zigzag nanotubes, the band gap lies in the range of 0.76–1.98 eV going from (9,0) to (26,0) sizes. The maximum gap is going towards the indirect band gap obtained for the β monolayer, reported previously, about 2.26 eV [16]. Furthermore, it was found that there is a net charge transfer from silicon to sulfur atoms in all the nanotubes studied. For nanotubes, the amount of charge transfer decreases marginally with size below 0.1 electrons. As the nanotube becomes wider, the charge values approach those of the SiS β monolayer, being about 0.33 electrons in the Mulliken scheme, because the curvature is much reduced for larger sizes.

*Focusing on the (6,6) β nanotube*. Stability at different temperatures was investigated using molecular dynamics simulations for the two most stable β SiS nanotubes, i.e., the (6,6) and (16,0) NTs. The zigzag (16,0) NT was found to be unstable at room temperature and eventually broke down, whereas the (6,6) nanotube remained stable. Consequently, we focused on the electronic properties of the armchair nanotube minimum, while the properties of the (16,0) zigzag nanotube are included in Appendix C for comparison.

**Figure 9:**
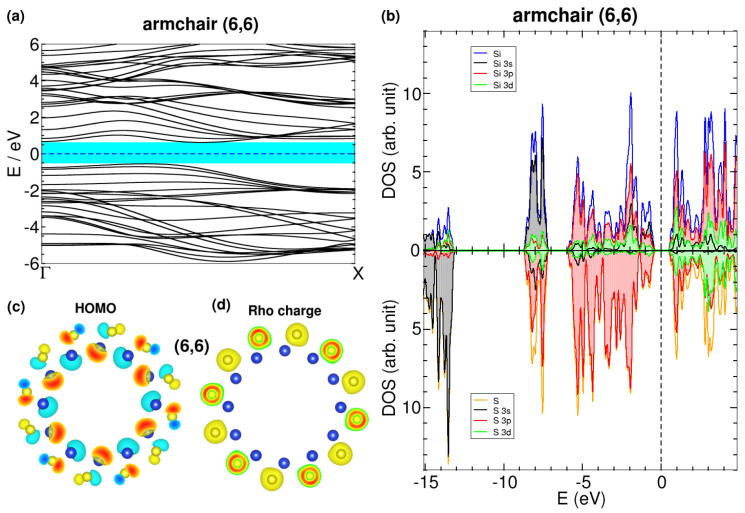
Electronic properties of the stable state (6,6) armchair β SiS nanotube: (**a**) band structure, (**b**) projected density of states on atom orbitals, (**c**) highest occupied molecular orbital (HOMO), and (**d**) charge density. Plotted contours in panels (**c**,**d**) are defined around 5% of the maximum positive and negative values inside, which are about ±5 × 10${}^{-3}$ e/Å${}^{3}$. The charge density is cut around some S atoms, where inside maxima are shown in red color.

**Figure A7:**
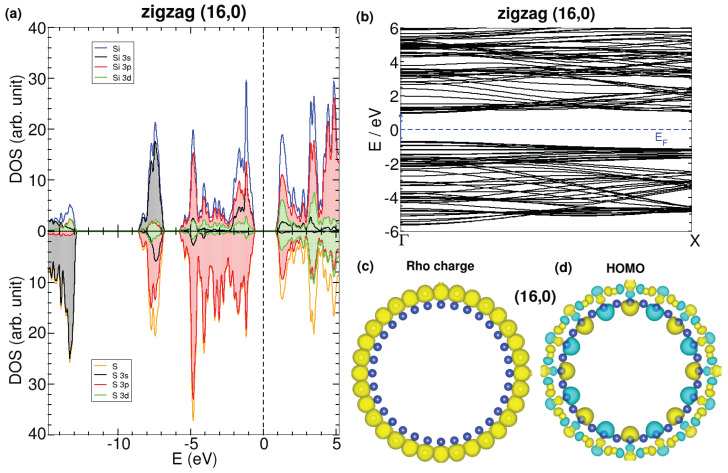
Electronic properties of the ground state (16,0) zigzag β SiS nanotube: (**a**) projected density of states, (**b**) band structure, (**c**) charge density, and (**d**) highest occupied molecular orbital (HOMO). Note the presence of a direct band gap in the electronic structure and the difference in electron density between Si and S atoms in (**c**).

The band structure of the (6,6) β SiS nanotube is shown in Figure 9a,b. Although the (6,6) nanotube is a semiconductor, its band gap is indirect with a value of 1.17 eV. The valence bands are degenerated at the *X* point of the Brillouin zone and split as they approach the Γ point. In contrast, the (16, 0) bands (included in Figure A7) have a direct band gap of 1.64 eV at the Γ point, and the bands are nearly flat close to the Fermi energy.

We next examined additional noteworthy electronic properties of the (6,6) minimum. Figure 9c shows the highest occupied molecular orbital (HOMO) for the (6,6) nanotube. It has *p*-type lobes on the sulfur atoms and *sp* lobes localized on the silicon ones, a localization in Si that is consistent with the projected density of states shown in Figure 9b. Finally, in Figure 9c,d, we observed the charge density for the (6,6) nanotube, with electrons being positioned around the sulfur atoms in the outer part of the nanotubes. The SiS nanotubes have a positive charge inside and a negative charge outside, following a pattern that might have practical applications. For example, by using electric fields, these tubes could be manipulated for electronic transport in the valence (outer) or conduction (inner) band, as discussed similarly for the photovoltaic proposals developed in imogolite nanotubes [35].

We last provided some comments on the experimental realization of SiS nanotubes. Currently, the synthesis of SiS layers in the *Pmma* and β phases remains a challenge and would require a bottom-up approach such as chemical vapor deposition (CVD) and molecular beam epitaxy (MBE) since these two phases have not been obtained in bulk as to allow for exfoliation. It is conceivable that SiS nanotubes could be produced via molecular beam epitaxy (MBE). However, the difficulty lies in the formation of the monosulfide phase rather than SiS${}_{2}$. One way to avoid the formation of SiS${}_{2}$ phases is by growing the nanotubes on certain templates, as has been done with other analogous chalcogenide tubes [7]. Hence, these challenges encountered when attempting to synthesize monosulfide silicon nanotubes have to be considered. Additionally, the nanotubes with the Pmma phase would require metallic seeds to allow for the layer structure to bend, while the β ones could be naturally obtained without seeds due the inner strain between both Si–S sides in the hexagonal buckled layer. It is noteworthy that blue phosphorene in the beta phase, isolectronic to the β nanotubes and with the same structure, was already synthesized on Au substrates using MBE under certain thermal conditions [36]. Note that nanotubes with negative strain energy are today synthesized using colloidal techniques [13]. Furthermore, monosulfides such as SnS are already found as nanotubes and nanoscrolls due to the misfit between close SnS${}_{2}$ and SnS layers [7], with a breaking of symmetry in the c direction, which is already implicit in the β SiS layers. Monosulfide nanotubes build upon the previous research on nanotubes, such as in the investigation of quaternary misfit layered compound (MLC) Y${}_{x}$La${}_{1-x}$S-TaS${}_{2}$ nanotubes (NTs), which were successfully synthesized via the chemical vapor transport technique, allowing for the creation of stable nanotubes with various compositions throughout the entire Y-La composition range [8], highlighting their suitability for diverse applications. On the other hand, the silicon monosulfide nanotubes exhibited compelling optical properties in the near-infrared region, alongside their combined thermal stability, which presents opportunities for novel applications. The gaps of the β nanotubes are in the right range, and a further look at the transition probabilities would be needed; however, these tubes are not being found the most stable. Anyhow, the Pmma nanotubes have already shown interesting properties as described in Figure 6. These findings suggest some further ideas that need to be considered for future research on the synthesis due to the interest in optical properties of these SiS nanotubes.

## 4. Conclusions

The present work reports on the study of SiS nanotubes derived from two different SiS phases. Our results show that both types of nanotubes are stable at room temperature, with the β phase armchair nanotube being even more stable that the corresponding layer phases. We found that the *Pmma* SiS nanotubes exhibit a dimerization effect, leading to semiconductor tubes, while the regular tubes are metallic. Control of the dimerization could thus lead to a change in electronic conductivity. On the other hand, the β SiS armchair nanotubes exhibit negative charge localized outside the tube, which could be of interest for electronic transport applications. Overall, our findings suggest that both types of SiS nanotubes are promising for optoelectronic applications due to the effects of curvature on valence and conduction electrons. The synthesis of these nanotubes still remains a challenge, and further research is needed to explore their full potential for applications.

## Figures and Tables

**Figure A1:**
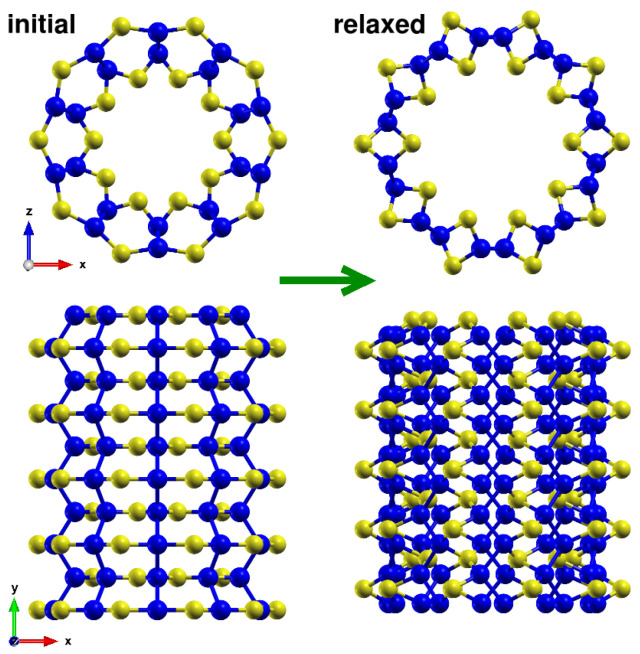
Comparison of the initial guess and relaxed structure of the (10, 0) *Pmma* nanotube showing the significant changes during relaxation. Atoms are following the color code given in the main text. The initial guess structure is less stable than the relaxed structure, which adopts a different conformation with rotated silicon lines. The relaxed structure is still in nanotube form but with a different structure from the Pmma phase.

**Figure A2:**
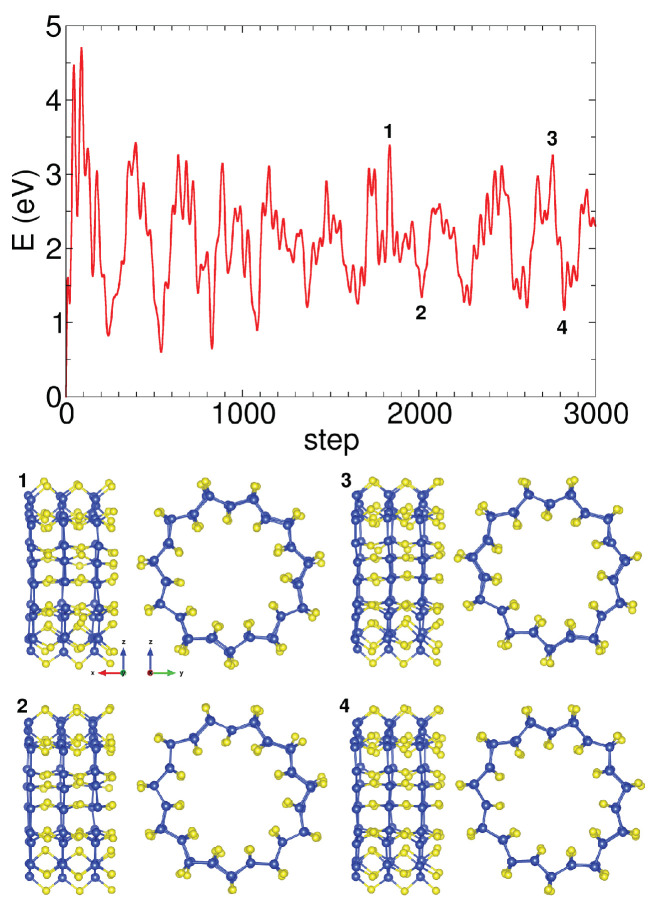
Molecular dynamics simulation of the (0,11) *Pmma* SiS nanotube showing the total energy versus steps. Snapshots of the nanotube at different steps are shown to illustrate the dynamics.

**Figure A3:**
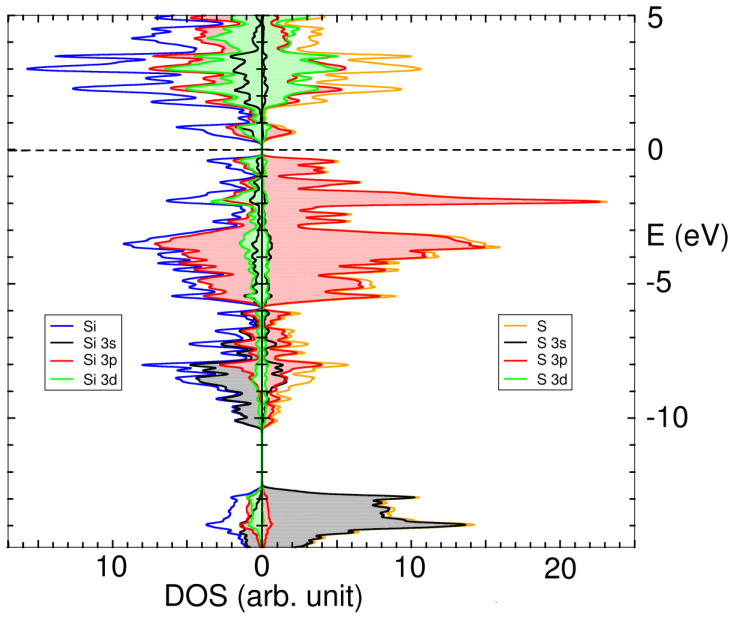
Density of states (DOS) of the dimerized (0,10) *Pmma* SiS nanotube projected onto the atomic orbitals of Si and S atoms, including *s*, *p*, and *d* states. Note that there is a non-negligible contribution of *d*–Si states around the Fermi energy.

**Figure A4:**
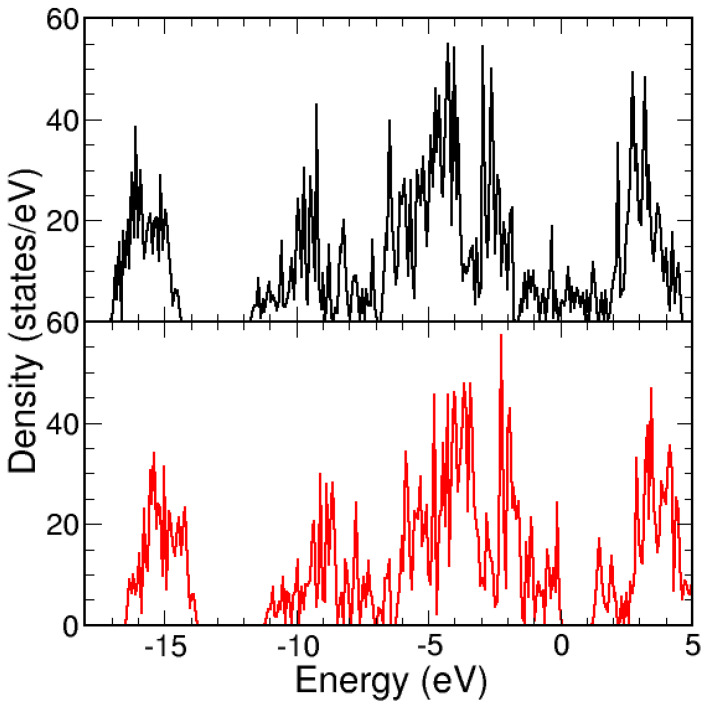
Density of states (DOS) for the (0,10) Pmma nanotube using a hybrid functional. Upper panel: DOS of the regular nanotube, which remains metallic. Lower panel: DOS of the dimerized nanotube, which becomes a semiconductor. The results are in agreement with the GGA calculations presented in the main text.

**Figure A5:**
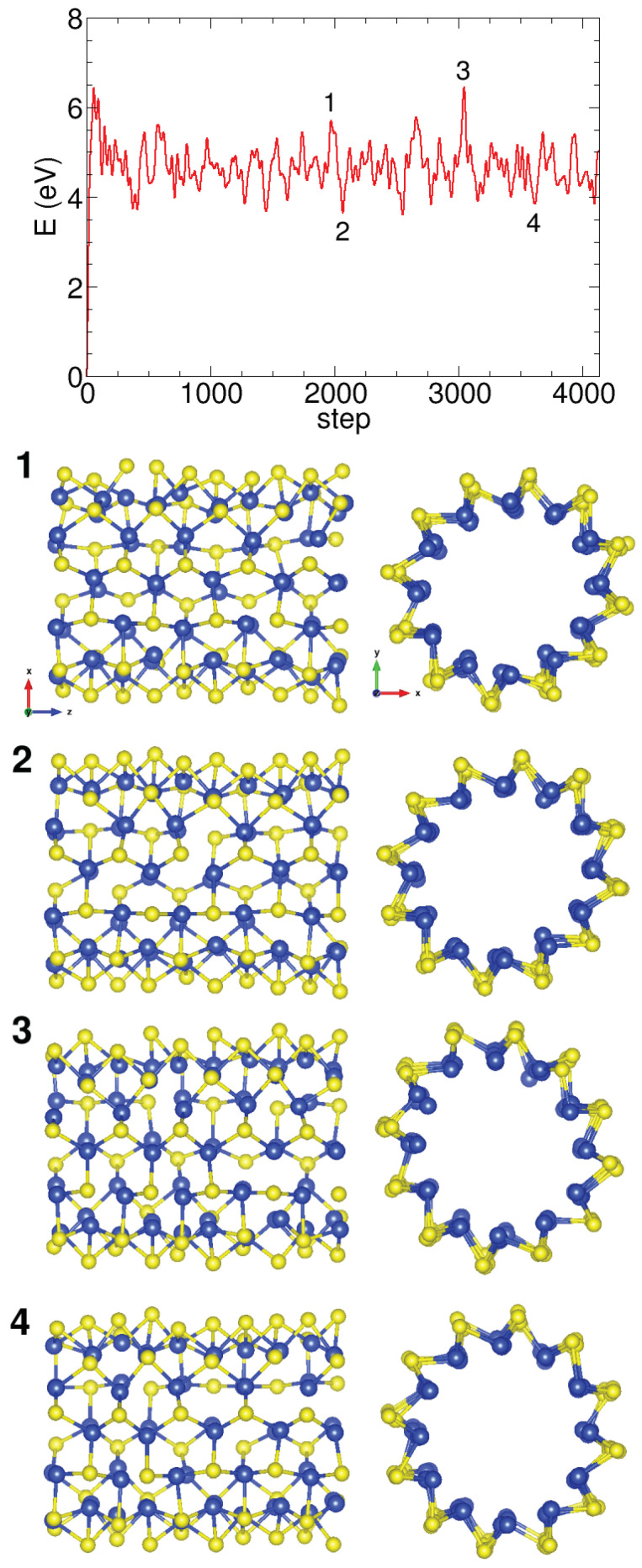
Energy versus step in the molecular dynamics simulation of the (6,6) armchair β SiS nanotube. Snapshots of four different steps were shown to illustrate the dynamics.

**Figure A6:**
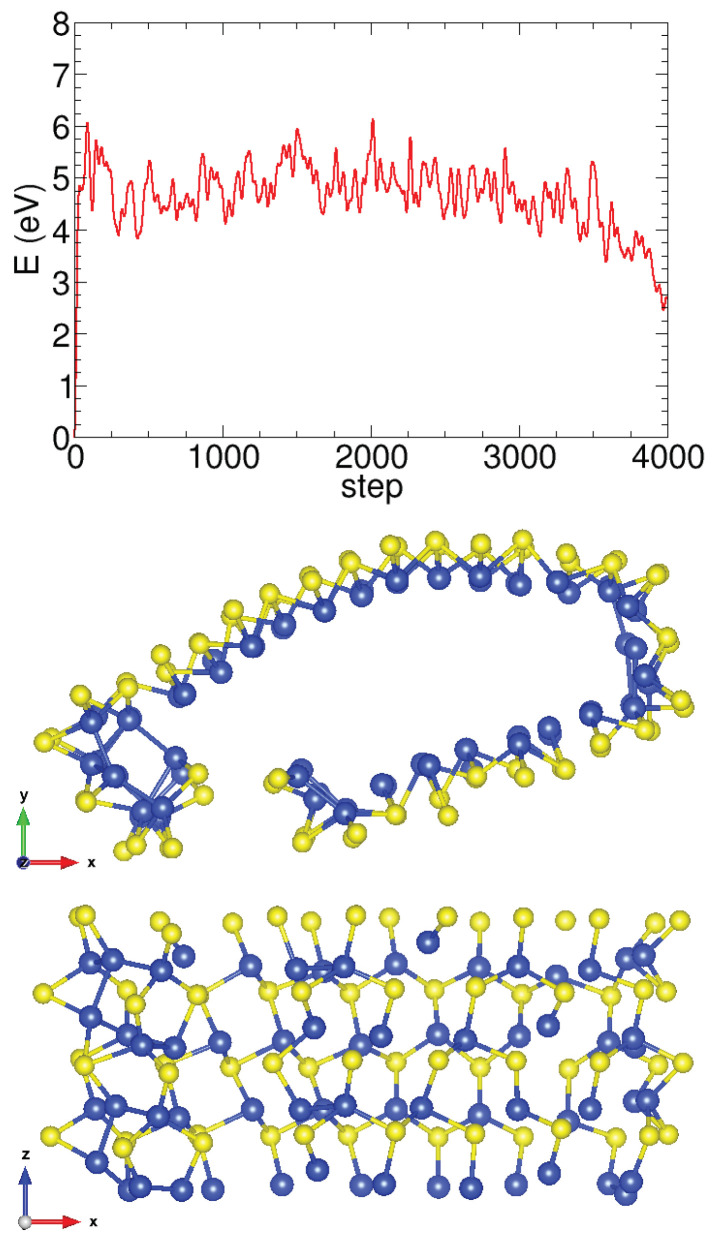
Total energy versus simulation step in the molecular dynamics study of the (16,0) zigzag β SiS nanotube. The two perspectives of a snapshot taken with a large number of steps show that as the simulation progresses, the nanotube becomes increasingly flat and eventually breaks.

## Data Availability

Data are contained within the article.

## Abbreviations

The following abbreviations are used in this manuscript:

TMDCs	transition metal dichalcogenides
DFT	density functional theory
GGA	generalized gradient approximation
NT	nanotube

## Appendix A. Pmma SiS Nanotubes

### Appendix A.1. Reconstruction in (n,0) Nanotubes

Upon relaxations, the (n,0) SiS nanotubes undergo significant structural changes, as shown in Figure A1. The relaxed structures of the (n,0) tubes are less stable compared to the corresponding (0,n) nanotubes rolled in the perpendicular direction. The initial (n,0) tubes contain silicon lines that rotate at right angles during relaxation, resulting in a different structure than the *Pmma* phase. The large distortions observed during relaxations are attributed to the preference of Si atoms for tetrahedral coordination. In fact, the relaxed structures of the (n,0) Pmma tubes are reconstructed in the silicene structure of the thick SiS layers, which is beyond the scope of this study [17]. In any case, it is important to note that the relaxed geometry can still retain the nanotube form.

### Appendix A.2. Molecular Dynamics

Molecular dynamics simulations of (0,10) *Pmma* SiS nanotube reveal its stability at room temperature. The atoms move, oscillating slightly around the equilibrium positions, as shown in Figure A2, so that the *Pmma* geometries are preserved during the simulations in the (0,n) nanotubes.

### Appendix A.3. DOS of Dimerized Nanotube

The partial DOS decomposed in elements is shown in Figure A3. This quantity is interesting to follow the element contributions in several energy regions.

## Appendix B. Tests Using Hybrid Functionals

It appears that metallic tubes undergo a distortion to open a gap. To confirm that the nanotube behavior is independent of the exchange correlation scheme used, we conducted hybrid calculations HSE06 [37]. Since GGA calculations show metallic behavior for Pmma nanotubes, it could be expected that, using a hybrid functional, a gap could even lead to gap opening. Additionally, the PBE exchange-correlation functional is known to underestimate band gaps, so we had to check when the gap opening by including distortions happens by performing hybrid functional calculations. We tested the regular and distorted (0,10) Pmma nanotubes using hybrid calculations. The corresponding DOS are shown in Figure A4. The upper panel shows that the DOS of the regular tube remains metallic when using hybrid calculations. Furthermore, hybrid calculations in the lower panel indicate that the gap for the distorted structure just slightly increases with respect to the GGA results. Thus, the effect of including hybrid functionals can be neglected when discussing the gap opening and related properties in the main text.

## Appendix C. *β* SiS Nanotubes

We also performed molecular dynamics calculations at room temperature for the two stable structures of the armchair and zigzag β nanotubes using the SIESTA method with a Nose thermostat, a Nose mass of 10 Ry fs${}^{2}$, and a time step of 1 fs to ensure the stability of the relaxed structures with temperature. The (6,6) armchair nanotube shows oscillations in the total energy and atomic postions around the equilibrium positions of the β structure, as depicted in Figure A5. Conversely, the (16,0) zigzag nanotube is found to be unstable at room temperature and eventually breaks, as shown in the snapshot of Figure A6. We observed that the energy continuously decreases as the simulation progresses, indicating nanotube rupture at room temperature.

In order to compare with those for the (6,6) armchair minimum in the main text, the electronic properties of the (16,0) zigzag SiS nanotube are shown in Figure A7. The projected density of states for the (16,0) nanotube is similar to that of the (6,6) nanotube. The sulfur *s* states are deep in energy with a negligible contribution of silicon. The *s* states of silicon are at the same energy as the *p* states of sulfur with a slight *d* contribution. Finally we found a range of energies with *p* states of sulfur and silicon close to the Fermi energy, where the *d* states of silicon have a non-negligible contribution.
